# Genomic Characterization of *Lactobacillus delbrueckii* Strains with Probiotics Properties

**DOI:** 10.3389/fbinf.2022.912795

**Published:** 2022-06-06

**Authors:** Luís Cláudio Lima De Jesus, Flávia Figueira Aburjaile, Thiago De Jesus Sousa, Andrei Giacchetto Felice, Siomar De Castro Soares, Luiz Carlos Junior Alcantara, Vasco Ariston De Carvalho Azevedo

**Affiliations:** ^1^ Department of Genetics, Ecology and Evolution, Federal University of Minas Gerais, Belo Horizonte, Brazil; ^2^ Department of Preventive Veterinary Medicine, Federal University of Minas Gerais, Belo Horizonte, Brazil; ^3^ Department of Immunology, Microbiology and Parasitology, Federal University of Triângulo Mineiro, Uberaba, Brazil; ^4^ Flavivirus Laboratory, Instituto Oswaldo Cruz, Fundação Oswaldo Cruz, Rio de Janeiro, Brazil

**Keywords:** comparative genomics, core genome, probiogenomics, GIT stress response, bacteriocins, immunoregulatory proteins

## Abstract

Probiotics are health-beneficial microorganisms with mainly immunomodulatory and anti-inflammatory properties. *Lactobacillus delbrueckii* species is a common bacteria used in the dairy industry, and their benefits to hosting health have been reported. This study analyzed the core genome of nine strains of *L. delbrueckii* species with documented probiotic properties, focusing on genes related to their host health benefits. For this, a combined methodology including several software and databases (BPGA, SPAAN, BAGEL4, BioCyc, KEEG, and InterSPPI) was used to predict the most important characteristics related to *L. delbrueckii* strains probiose. Comparative genomics analyses revealed that *L. delbrueckii* probiotic strains shared essential genes related to acid and bile stress response and antimicrobial activity. Other standard features shared by these strains are surface layer proteins and extracellular proteins-encoding genes, with high adhesion profiles that interacted with human proteins of the inflammatory signaling pathways (TLR2/4-MAPK, TLR2/4-NF-κB, and NOD-like receptors). Among these, the PrtB serine protease appears to be a strong candidate responsible for the anti-inflammatory properties reported for these strains. Furthermore, genes with high proteolytic and metabolic activity able to produce beneficial metabolites, such as acetate, bioactive peptides, and B-complex vitamins were also identified. These findings suggest that these proteins can be essential in biological mechanisms related to probiotics’ beneficial effects of these strains in the host.

## Introduction

*Lactobacillus delbrueckii* is the type species of *Lactobacillus* genus after a new proposed taxonomic reclassification that divided this genus into 25 new, based on genetic and phylogenetic analysis associated with ecological and metabolic properties (Zheng et al., 2020). This Lactic Acid Bacteria (LAB) member comprises gram-positive, rod-shaped, facultatively anaerobic, and acid-resistant microorganisms, which occupy diverse carbohydrate-rich environments with final fermentative metabolism-derived lactic acid production (Salvetti et al., 2012; Duar et al., 2017). This species includes mainly two subspecies: *bulgaricus* and *lactis,* both with high importance in industrial fermented dairy products (primarily yogurt and cheeses production) and biotherapeutics approaches (Hao et al., 2011; El Kafsi et al., 2014; Santos Rocha et al., 2014).

Some studies have been characterizing the *L. delbrueckii* strains as probiotics based on their ability to resist gastrointestinal tract (GIT) stressors (Ferreira et al., 2013), pathogens inhibition (De Jesus L. C. L. et al., 2021), and anti-inflammatory effects mainly focused on GIT disease treatment, such as colorectal cancer (Wan et al., 2014), ulcerative colitis (Santos Rocha et al., 2014), and intestinal mucositis (De Jesus et al., 2019). In addition, pre-clinical therapeutical applications of these microorganisms to other pathological conditions, such as arthritis (Kano et al., 2013), depression (Qiu et al., 2021), and diabetes (Hallajzadeh et al., 2021), have also been reported. Among this species, *L. delbrueckii* subsp. *lactis* CIDCA 133 is the best-characterized probiotics strain whose beneficial characteristics and safety aspects have been widely evaluated by *in vitro* and *in vivo*, as well as *in silico* analysis, for example, its ability to inhibit *Escherichia coli*, *Bacillus cereus*, *Citrobacter rodentium*, and *Salmonella* Typhimurium pathogens; immunomodulation by inhibition of NF-κB signaling pathway; tolerance to high concentrations of bile salts; no hemolytic or mucin degradation activity, and no adverse effects to clinical and histopathological mice parameters (Rolny et al., 2016; Hugo et al., 2017; De Jesus et al., 2019; De Jesus L. C. L. et al., 2021; De Jesus LCL. et al., 2021; Barroso et al., 2022).

Although most studies focus on the effect and action mechanism of viable probiotic strains, there is a growing interest in applying probiotics as microbiologically non-viable but immunologically active products. This would be more viable and safer for probiotic applications in clinical practice due to safety concerns regarding this active metabolic form favoring the risk of bacterial translocation (Moradi et al., 2020; Teame et al., 2020). Some studies have evaluated the inactivation of these microorganisms or products derived from them in different inflammation models and obtained similar results to their metabolically active form (Sang et al., 2013; Nakai et al., 2021; Trindade et al., 2021).

According to Hill et al. (2014), probiotics are defined as “live microorganisms that confer a health benefit on the host when administered in adequate amounts.” However, it should also be highlighted that most of these beneficial effects attributed to probiotics are strain-dependent, revealing that individual characteristics of the strains provide relevant data for the development of effective probiotic products and facilitate individualized or personalized use for clinical applications (Bubnov et al., 2018; McFarland et al., 2018). This strain-specific property was more substantially related by Rocha et al. (2012) that, when screening 57 dairy *L. delbrueckii* strains, observed that the immunomodulation levels of these bacteria varied depending on the strain. Among the 37 *L. delbrueckii* subsp. *bulgaricus* and 20 *L. delbrueckii* subsp. *lactis* strains tested, the most effective immunomodulators strains belong to the subsp. *lactis* (Rocha et al., 2012), including CNRZ327 and CNRZ333 strains.

Individual biological properties of probiotic strains may be related to a high degree of variation in their genomic content. Thus, studies at the genomic level can provide insights into the main genetic factors and molecular mechanisms associated with the probiotic features of these microorganisms, such as GIT survival, pathogens inhibition, and immunoregulation (Ventura et al., 2012; Salvetti and O,Toole, 2018; Castro-López et al., 2021). Probiotics studies using the genome approach have been performed to identify genetic factors involved with features of different potential probiotics strains, such as *Lactobacillus helveticus* (Fontana et al., 2019), *Lactiplantibacillus plantarum* (Zhang et al., 2018), *Pediococcus sp.* (Wanna et al., 2021), *Bifidobacterium sp*. (Duar et al., 2020), *Enterococcus sp*. (Hussein et al., 2020), *Lactococcus lactis* (Oliveira et al., 2017)*,* among others. In this context, the comparative analysis proves to be an essential tool in probiogenomics, contributing to further exploring the diversity and evolutionary relationship of species (Sun et al., 2015), and identifying and comparing the gene repertoire in different strains (Fontana et al., 2019) and the relationship of these molecules with reported probiotics effects of these bacteria on the host (Papadimitriou et al., 2015; Sun et al., 2015).

Although the importance of *L. delbrueckii* strains in the food industry, few studies have focused on genomic studies of *L. delbrueckii* probiotics strains regarding their host health benefits (El Kafsi et al., 2014; Sun et al., 2015; Kanmani et al., 2018; De Jesus L. C. L. et al., 2021). Thus, this study carried out a comprehensive functional gene characterization of *L. delbrueckii* species with reported probiotics effects, which may be associated with the specific host health benefits of these strains reported phenotypically, and provide a better comprehension of their probiotics features.

## Materials and Methods

### Genome Data

Nine genomes of *L. delbrueckii* strains with reported probiotics properties in the literature (Savino et al., 2011; Santos Rocha et al., 2014; Li et al., 2015, 2017, 2020; Kanmani et al., 2018; Usui et al., 2018; De Jesus et al., 2019; El-Khadragy et al., 2019) were downloaded from the NCBI database (Table 1). The genome assemblies were evaluated by QUAST 5.0.2 (Gurevich et al., 2013) and BUSCO v4.0.6 software (Benchmarking Universal Single-Copy Orthologs) (Simão et al., 2015). In addition, all genomes were annotated using the Prokaryotic Genome Annotation System (Prokka) v1.14.5 software (Seemann, 2014).

**TABLE 1:** Genome features of *Lactobacillus delbrueckii* strains used in this study.

Strain	Genome Access	Size (Mb)	GC%	CDS	Source	Probiotic Property	References
LJJ	NZ_CP049052.1	1.89	49.50	1,604	Dairy products	Acid tolerance mechanism	Li et al. (2020)
KLDS1.0207	NZ_CP032451.1	1.87	49.80	1,607	Dairy products	Alleviation of lead (Pb) toxicity	Li et al. (2017)
DSM 20080	NZ_CP019120.1	1.87	49.80	1,680	Environment	Oxidative stress modulation on *S. mansoni*-infected mice	El-Khadragy et al. (2019)
2038	NC_017469.1	1.87	49.70	1,792	−−−	Microbiota regulation in aging mice	Usui et al. (2018)
ATCC 11842	NC_008054.1	1.86	49.70	1,683	Bulgarian Yogurt	Osmotic tolerance mechanism	Li et al. (2015)
TUA4408L	NZ_CP021136.1	2.01	49.90	1,801	Sunki-zuke	Immunomodulatory activity on rotavirus infection	Kanmani et al. (2018)
DSM20074	NZ_CP018615.1	1.95	49.60	1,721	Environment	Pathogen inhibition	Savino et al. (2011)
CNRZ327	GCF_000751695.1	2.11	49.60	1,525	Cheese	Anti-inflammatory effect on DSS-induced Colitis	Santos Rocha et al. (2014)
CIDCA 133	CP065513	2.13	49.59	1,921	Raw cow’s milk	Anti-inflammatory effect on 5-FU-induced Mucositis	De Jesus et al. (2019)

### Pan-Genome Analysis

This study used the BPGA (Bacterial Pan Genome Analysis) pipeline for performance pan-genome (Chaudhari et al., 2016). The genome sequences were submitted in FASTA format to Orthofinder software to predict orthologs genes (Emms and Kelly, 2019), using default parameters with a *p*-value cut-off of 1E^−5^. This software bases its inference method on OrthoMCL (Li et al., 2003) through the hybrid Markov Clustering algorithm (Enright, 2002), which computes sequence similarities with BLAST and then uses the MCL clustering algorithm to identify clusters of highly connected sequences. After this process of predicting orthologous genes, through in-house scripts, these genes were classified according to the subsets of the pangenome, being divided into the core genome, shared, and singletons. For the development of the pangenome, after the classification process in its subsets, in-house scripts were used to estimate what would be the fixed parameters of the Heap Law (Soares et al., 2013; Guimaraes et al., 2015) and the Utterance of the Least Squares Principle (for core genome subsets and singletons). For genetic contexts, we can represent Heap’s Law according to the formula n = k *N γ, which (n) would be the value for the number of genes for a given number of genomes (N); and then k and γ can be considered as free parameters. By this law, γ can be calculated as α = 1—γ, so when α > 1 (γ < 0), the pangenome is called closed, which means that there is no increase, or there is no significant increase, of genes when more genomes of the studied organism are sequenced. If α < 1 (0 < γ < 1), suggests the pan-genome of the probiotic strains is open, which indicates that there is an increase in the number of genes when more genomes are sequenced. The Least Squares Principle Statement can be represented by the formula n = k * exp [−x/t] + tgθ, where (n) is also the number of genes, and k, t, and tgθ are considered as parameters free. With the result of this law, we were able to estimate, based on the number of singletons added to each new sequencing, how many genomes are still needed for the core genome of the studied group to reach stability.

### Functional Annotation of Pan-Genome

Clusters of Orthologous Groups (COGs) for core genes, accessory genes, and singletons (first unique genes of the strains) were obtained using the eggNOG-mapper v2 web tool (http://eggnog-mapper.embl.de/) (E-value < 0.001) (Cantalapiedra et al., 2021). Furthermore, a complementary functional annotation analysis was carried out using the Kyoto Encyclopedia of Genes and Genomes (KEGG) through the KEGG Mapper/BLASTKOALA tool (https://www.kegg.jp/blastkoala/) (Kanehisa et al., 2016).

### Prediction of Genes Related to Antibacterial Activity

Proteins involved in antibacterial activity were also evaluated across the probiotics *L. delbrueckii* genomes. For this purpose, genes coding bacteriocins were predicted through BAGEL4 (http://bagel4.molgenrug.nl/) (van Heel et al., 2018). The bacteriocins-encoding genes’ distribution among the genomes was visualized through a heatmap of presence and absence. Furthermore, core proteins producing other antimicrobial compounds, such as hydrogen peroxide and organic acids, were investigated using the KEGG Mapper/BLASTKOALA tool (Almeida et al., 2021).

### Identification of Gastrointestinal Tract Stress Response Genes and Proteolytic Enzymes in Core Genome

Identification of core proteins of probiotics *L. delbrueckii* strains related to GIT stress response (acid and bile) and proteolytic activity were manually predicted through Prokka–derived annotation, based on previous studies (Liu et al., 2010; Papadimitriou et al., 2016; De Jesus LCL. et al., 2021).

### Prediction of Metabolic Pathway-Related Genes in Core Genome

The presence of genes involved in metabolic pathways related to carbohydrate metabolism, lactate, short-chain fatty acids (SCFAs), and vitamin B biosynthesis was predicted using the BioCyc database (https://biocyc.org/) (Karp et al., 2019). The genomes of *L. delbrueckii* subsp. *lactis* DSM 20072 (Genome access: NZ_CP022988.1) and *L. delbrueckii* subsp. *bulgaricus* ATCC 11842 (Genome access: NC_008054.1) strains were used for this comparative analysis. Furthermore, the carbohydrate-active enzymes (CAZymes) families were predicted through the Carbohydrate-Active Enzyme (CAZy) database (http://www.cazy.org/) (Cantarel et al., 2009).

### Interaction of Core Proteins of *Lactobacillus delbrueckii* Strains With Human Immune Proteins

To evaluate the potential biological interaction between core proteins of *L. delbrueckii* probiotic strains and human immune proteins, first, the subcellular localization of proteins identified in the core genome was predicted using SurfG+ software (Barinov et al., 2009). Second, the core proteins were predicted for their ability to be an adhesin calculated by SPAAN software (score >0.7) (Sachdeva et al., 2005). After, immune protein sequences related to the inflammation pathways (TLR2/4-MAPK, TLR2/4-NF-κB, and NOD-like receptor signaling pathways) were mapped and obtained from KEGG pathways and UniProt (UP000005640), respectively (Supplementary Table S1). Finally, the protein-protein interaction was conducted in the InterSPPI v2 web server (http://zzdlab.com/InterSPPI/) (Lian et al., 2019). Graphical analysis of resulting interaction networks (minimum score: 0.9765; specificity: 0.99) was performed by Cytoscape v3.9.0 software (Shannon, 2003).

## Results

### Genome Features of Probiotics *Lactobacillus delbrueckii* Strains

The probiotics group of *Lactobacillus delbrueckii* strains evaluated in this study is mainly formed by the subspecies *bulgaricus* (n = 5) (LJJ, KLDS1.0207, DSM 20080, 2038 and ATCC 11842), *delbrueckii* (n = 2) (TUA4408L, DSM20074), and *lactis* (n = 2) (CNRZ327 and CIDCA 133). The strains were mainly isolated from dairy environments, including cheeses, yogurts, and fermented milk. The genome evaluation of these nine strains revealed a genome size and GC content average of 1,951 ± 0.10 Mb and 49.69 ± 0.12%, with 1,664 ± 0.09 protein-coding sequences (CDS) (Table 1).

### Pan-Genome Analysis

**FIGURE 1:**
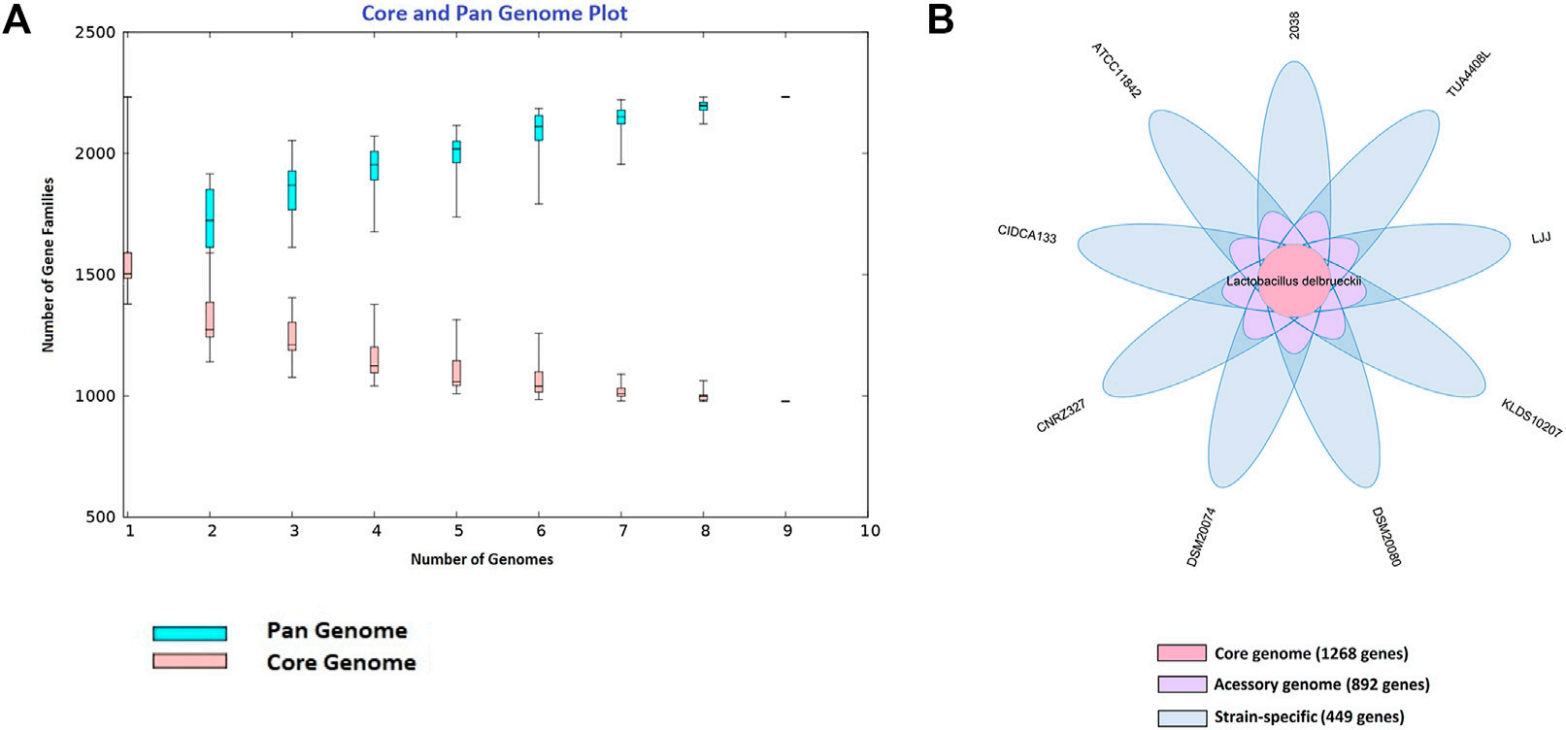
Pan-genome of probiotics *L. delbrueckii* strains. **(A)** Curve development of pan (blue color) and core (salmon color) genome (α = 0.83). The number of gene families is plotted in function of the genome number. **(B)** Venn diagram illustrating the core-genome size (center part), accessory genomes (around the center), and unique genes for each strain (extreme values).

The pan-genome (total gene repertoire) obtained through BPGA with nine probiotics *L. delbrueckii* strains is composed of a total of 2,609 genes (Figure 1A), of which 1,268 (48.60%) belong to the core genome (number of genes shared by all strains), 892 genes (34.18%) to the accessory genome (genes shared by two or more strains), and 449 (17.20%) are strain-specific (uniques) (genes present in a single strain) (Figure 1B). Furthermore, the CIDCA 133, DSM20074, and CNRZ327 strains presented the highest exclusive genes, with 102, 76, and 69 genes, respectively, followed by TUA4408L (53 genes), 2038 (47 genes), ATCC11842 (39 genes), KLDS10207 (32 genes), LJJ (16 genes), and DSM20080 (15 genes).

According to the curve generated for these nine genomes based on Heap’s Law and leats-square fit of the exponential regression decay, the number of genes families in the pan-genome increased with the addition of each other genome (n = 1,848.134*n^0.156), suggesting that the pan-genome of probiotics *L. delbrueckii* strains remains open. For the subsets of the core genome and singletons developed by the Utterance of the Least Squares Principle, it can be observed a value of tgθ of approximately 1,182 genes (n = 465.995 * exp [−x/4.839] + 1182.675) for the core genome, and a value of approximately 24 (n = 219.676 * exp [−x/4.356] + 24.813) for the strain-specific. This result shows that at each new sequencing, 24 new genes are added to this pangenome, and it is expected that the core genome will stabilize when it reaches around 1,182 genes (Figure 1A).

### Functional Annotation of Gene Families

**FIGURE 2:**
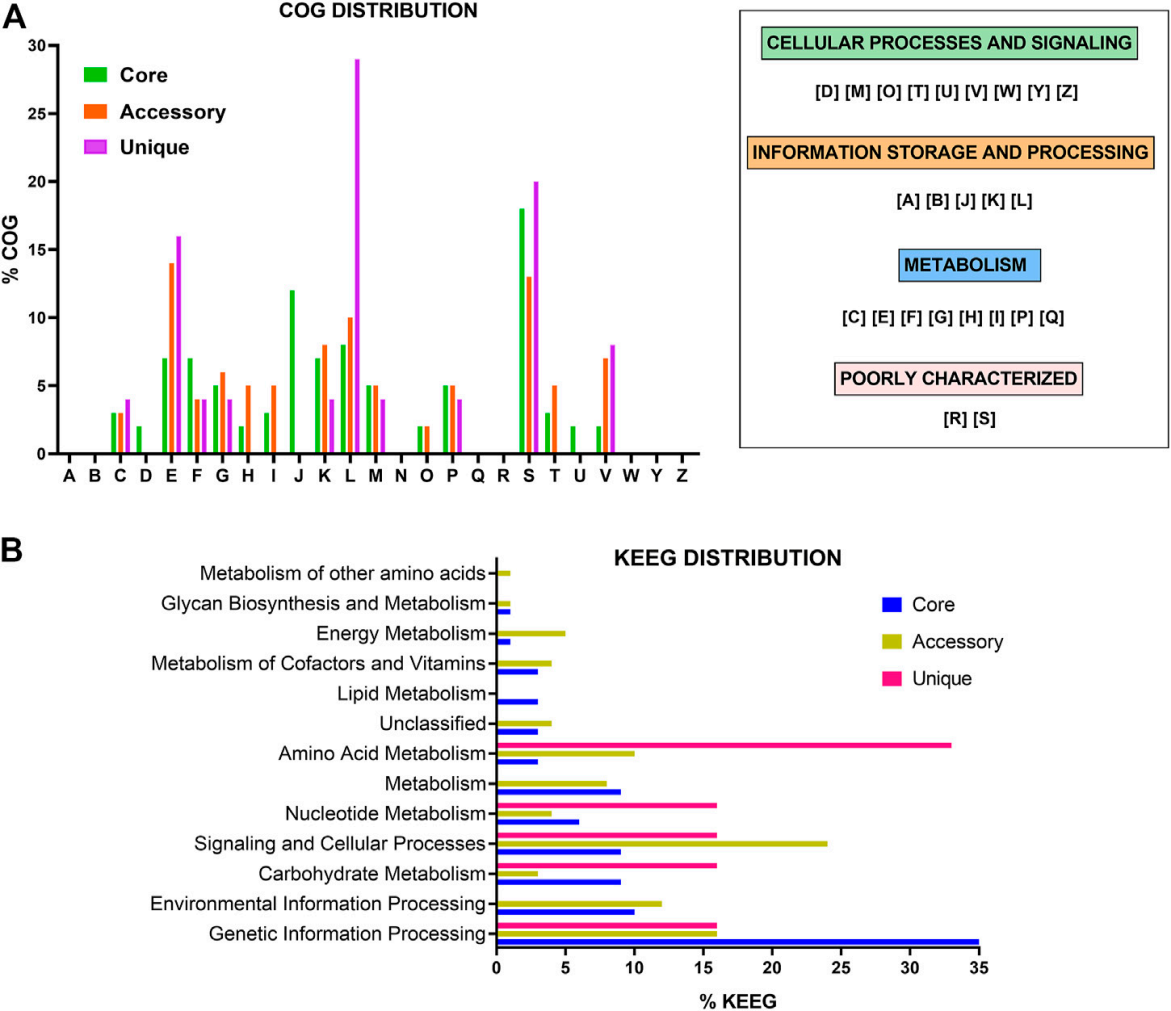
Functional analysis of gene families of probiotics *L. delbrueckii* strains. Distribution of COG **(A)** and KEGG **(B)** functional categories in the core, accessory, and unique genes. COG categories: [A] RNA processing and modification, [B] Chromatin structure and dynamics, [C] Energy production and conversion, [D] Cell cycle control, cell division, chromosome partitioning, [E] Amino acid transport and metabolism, [F] Nucleotide transport and metabolism, [G] Carbohydrate transport and metabolism, [H] Coenzyme transport and metabolism, [I] Lipid transport and metabolism, [J] Translation, ribosomal structure and biogenesis, [K] Transcription, [L] Replication, recombination and repair, [M] Cell wall/membrane/envelope biogenesis, [N] Cell motility, [O] Post-translational modification, protein turnover, and chaperones, [P] Inorganic ion transport and metabolism, [Q] Secondary metabolites biosynthesis, transport, and catabolism, [R] General function prediction only, [S] Function unknown, [T] Signal transduction mechanisms, [U] Intracellular trafficking, secretion, and vesicular transport, [V] Defense mechanisms, [W] Extracellular structures, [Y] Nuclear structure, and [Z] Cytoskeleton.

Analysis of the COG distribution for the pan-genome revealed that many of the proteins in the core genome are related to “translation, ribosomal structure, and biogenesis” (12%), “replication, recombination and repair” (8%), “amino acid transport and metabolism” (7%), “nucleotide transport and metabolism” (7%), and “unknown function” (18%). The accessory genome presented COG terms related to “amino acid transport and metabolism” (14%), “replication, recombination and repair” (10%), “transcription” (8%), “defense mechanisms” (7%), and “unknown function” (13%). Finally, “replication, recombination and repair” (29%), “amino acid transport and metabolism” (16%), “defense mechanisms” (8%), and “unknown function” (18%) were the most common COGs terms related to unique genes (Figure 2A).

Furthermore, the KEGG annotation revealed that most genes in the core genome are related to “genetic information processing” (35%), followed by “environmental information processing” (10%), “carbohydrates metabolism” (9%), and “signaling and cellular processes” (9%). In the accessory genome, most of the genes were related to “signaling and cellular processes” (24%), “genetic information processing” (16%), “environmental information processing” (12%), and “amino acid metabolism” (10%) function. To unique genes, “amino acid metabolism” (33%), followed by “genetic information processing” (16%), “signaling and cellular processes” (16%), and “carbohydrates metabolism” (16%) were the most frequent categories (Figure 2B).

### Core Proteins Involved in Gastrointestinal Tract Stress Responses

In the core genome of *L. delbrueckii* probiotics strains, it was identified some genes encoding proteins that were previously reported to be involved in GIT stress response (acid and bile), including enolase, serine protease HtrA, ornithine decarboxylase, two-component sensor histidine kinase, chaperones (DnaK, DnaJ, GroeL), Na+/H+ antiporter NhaC, F0F1 ATP system genes, S-ribosylhomocysteine lyase, ATP-dependent ClpX protease, glycine/betaine ABC transporter permease, among others (Table 2).

**TABLE 2:** Predicted proteins identified in the core genome of probiotics *Lactobacillus delbrueckii* strains involved in acid and bile tolerance.

Locus Tag	Predicted Protein	Gene
OHNDKLAL_00510	Putative ornithine decarboxylase	*odcl*
OHNDKLAL_01838	UDP-galactopyranose mutase	*glf*
OHNDKLAL_02062	Pyruvate oxidase	*pox1*
OHNDKLAL_00195	Peptidase M13	*pepO*
OHNDKLAL_01467	Two-component sensor histidine kinase	*arlS*
OHNDKLAL_01763	Na+/H+ antiporter NhaC	*nhaC*
OHNDKLAL_00075	L-lactate dehydrogenase	*ldh*
OHNDKLAL_00088	S-ribosylhomocysteine lyase	*luxS*
OHNDKLAL_00120	Serine protease HtrA	*htrA*
OHNDKLAL_00179	Universal stress protein	*usp5*
OHNDKLAL_00196	Potassium transporter Kup	*kup*
OHNDKLAL_00262	Glutamine-hydrolyzing GMP synthase	*guaA*
OHNDKLAL_00321	CTP synthetase	*pyrG*
OHNDKLAL_00365	30S ribosomal protein S19	*rpsS*
OHNDKLAL_00482	Exopolyphosphatase	*ppx3*
OHNDKLAL_00483	Polyphosphate kinase	*ppk*
OHNDKLAL_00531	ATP-dependent Clp protease	*clpE*
OHNDKLAL_00534	Phosphoenolpyruvate--protein phosphotransferase	*ptsI*
OHNDKLAL_00540	GTP pyrophosphokinase	*yjbM*
OHNDKLAL_00557	Recombinase recA	*recA*
OHNDKLAL_00587	Glyceraldehyde-3-phosphate dehydrogenase	*gap*
OHNDKLAL_00588	Phosphoglycerate kinase	*pgk*
OHNDKLAL_00595	Phosphate acetyltransferase	*pta*
OHNDKLAL_00636	Acetate kinase	*ackA*
OHNDKLAL_00656	F0F1 ATP synthase subunit A	*atpB*
OHNDKLAL_00657	F0F1 ATP synthase subunit C	*atpE*
OHNDKLAL_00658	F0F1 ATP synthase subunit A	*atpF*
OHNDKLAL_00659	F0F1 ATP synthase subunit B	*atpH*
OHNDKLAL_00660	F0F1 ATP synthase subunit Alfa	*atpA*
OHNDKLAL_00661	F0F1 ATP synthase subunit gamma	*atpG*
OHNDKLAL_00662	F0F1 ATP synthase subunit beta	*atpD*
OHNDKLAL_00663	F0F1 ATP synthase subunit epsilon	*atpC*
OHNDKLAL_00733	ATP-dependent ClpX protease	*clpX*
OHNDKLAL_00759	Arginyl-tRNA synthetase	*argS*
OHNDKLAL_00785	Pyruvate kinase	*pyk*
OHNDKLAL_00833	ppGpp (guanosine 3'-diphosphate 5-' diphosphate) synthetase	*relA*
OHNDKLAL_00840	3-oxoacyl-ACP synthase	*fabH*
OHNDKLAL_01171	Glycine/betaine ABC transporter permease	*opuB*
OHNDKLAL_01283	Enolase	*eno*
OHNDKLAL_01301	Molecular chaperone DnaJ	*dnaJ*
OHNDKLAL_01302	Molecular chaperone DnaK	*dnaK*
OHNDKLAL_01303	Heat shock protein GrpE	*grpE*
OHNDKLAL_01333	30S ribosomal protein S2	*rpsB*
OHNDKLAL_01377	Asp23/Gls24 family envelope stress response protein	*yloU*
OHNDKLAL_01506	Dihydroorotate dehydrogenase	*pyrD*
OHNDKLAL_01584	Chaperonin GroEL	*groL*
OHNDKLAL_01585	Chaperonin GroES	*groS*
OHNDKLAL_01700	Phosphoglycerate mutase family protein	*pgm*
OHNDKLAL_01903	Oligoendopeptidase F	*pepF*
OHNDKLAL_02047	Glucosamine-6-phosphate deaminase	*nagB*

Italics represents the gene ID of predicted proteins related to GIT stress response.

### Core Genome of *Lactobacillus delbrueckii* Probiotics Strains Have Potential Genes Involved in Proteolytic Activity, Carbohydrates Metabolism, and Secondary Metabolic Product

The core genome of *L. delbrueckii* probiotic strains encodes various proteolytic enzymes essential for their growth, survival, and organoleptic properties of dairy products manufacturing. These enzymes including oligopeptide ABC transporters system (*oppD, oppC, oppF, oppA, oppB*), peptidases (*pepM, pepQ, pepT, pepO, pepR*), and proteinases (*PrtB, PrtM*). The peptidases mainly cleave substrates containing casein, methionine, proline, cysteine, leucine, serine, asparagine, and glutamate-derived peptides (Supplementary Table S2).

Carbohydrate metabolism was identified as essential enzymes related to glucose, fructose, sucrose, mannose, chitobiose, and galactose. These proteins include 6-phospho-beta-glucosidase, glucokinase, mannose-6-phosphate isomerase, and phosphoglucomutase. Furthermore, some genes related to the transport of cellobiose, mannose, fructose, and glucose carbohydrates were also identified, mainly related to the PTS system, the main carbohydrate active-transport system in bacteria (Supplementary Table S3). It was also determined that the most abundant carbohydrate-active enzymes (CAZy) gene families in the core genome of *L. delbrueckii* probiotics strains belong to glycosyltransferases (GTs) families (GT1, GT2, GT4, GT26, GT28, GT51) (n = 10), followed by glycoside hydrolases (GHs) (GH4, GH13, GH31, GH32, GH73) (n = 7), and carbohydrate-binding modules (CBMs) (CBM48) (n = 1), respectively.

Genes encoding proteins such as glucokinase, glucose-6-phosphate isomerase, glyceraldehyde 3-phosphate dehydrogenase, phosphoglycerate kinase, ribulose-phosphate 3-epimerase, pyruvate kinase, phosphoketolase, lactate dehydrogenase, and acetate kinase were also identified in the core genome of *L. delbrueckii* probiotics strains. These essential proteins are involved in the homofermentative or heterofermentative pathways, producing lactate or acetate. Genes involved in the biosynthesis of complex B vitamins were also predicted, including thiamine pyrophosphokinase (thiamine or vitamin B1), a riboflavin kinase (riboflavin or vitamin B2), dihydrofolate reductase (folate or vitamin B9), and cob(I)alamin adenosyltransferase (cobalamin or vitamin B12). No propionate or butyrate-related gene was identified in the core genome (Supplementary Table S4).

### Probiotics *L. delbrueckii* Strains Harbors Genes Related to Antibacterial Profile

**FIGURE 3:**
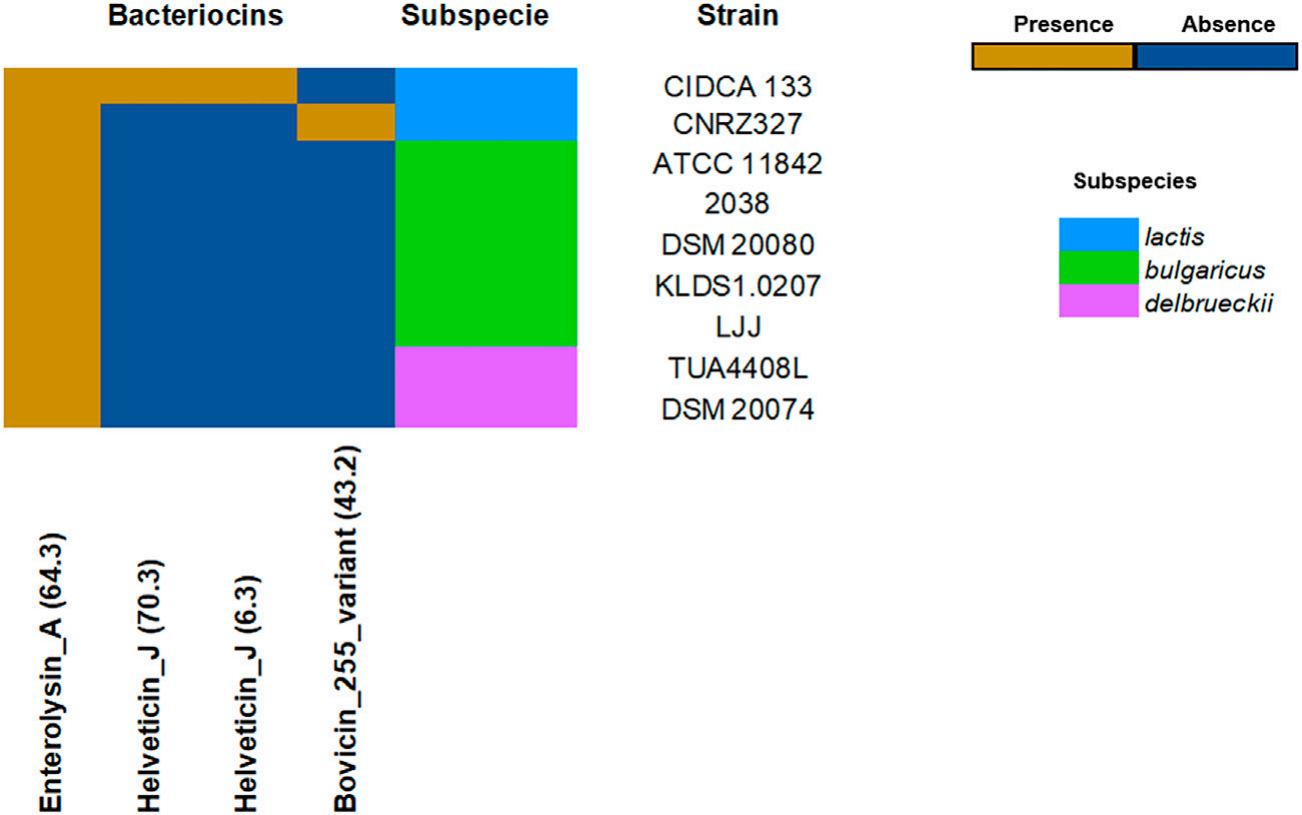
Bacteriocins profile across probiotics *L. delbrueckii* strains. The heatmap indicates the presence (dark yellow) or absence (dark blue) of bacteriocins-encoding genes.

The *L. delbrueckii* strains showed different profiles in terms of bacteriocins. Among all strains, subspecies *lactis* showed a greater diversity of bacteriocins in their genome, including enterolysin A, helveticin J, and bovicin_255. Few bacteriocins were found for the subspecies *bulgaricus*. The bacteriocins enterolysin A appears to be conserved in the species (Figure 3). Furthermore, it was identified in the core genome D-lactate dehydrogenase, L-lactate dehydrogenase, acetate kinase, L-lactate oxidase, glycolate oxidase, and pyruvate oxidase genes, which acts like crucial enzymes in the biosynthesis of organic acids (lactate and acetate), and hydrogen peroxide, respectively (Supplementary Table S5).

### Core Proteins With High Adhesin Profile Potentially Interacts With Human Immunomodulatory Proteins

**FIGURE 4:**
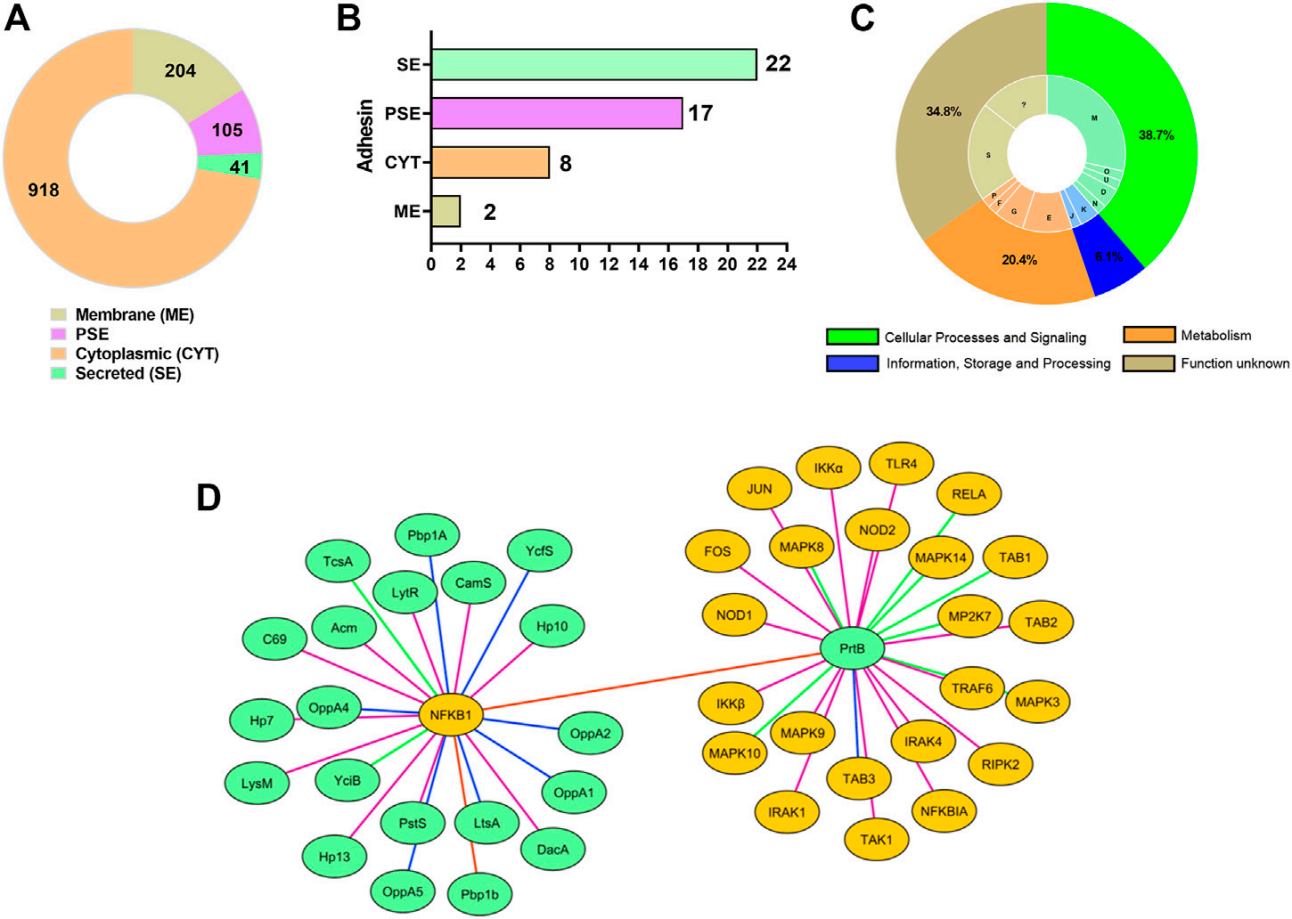
Core proteins of *L. delbrueckii* strains interact with human immune proteins involved in inflammatory pathways. **(A)** Subcellular localization of core proteins. **(B)** The number of core proteins with adhesin profile. **(C)** COG categories are assigned to core proteins with adhesin profiles. **(D)** Subnetwork mapping of *L. delbrueckii* core proteins (green circles nodes) interacting with human immune proteins (yellow, dark circle nodes). Different line colors indicates the interaction degree between the proteins, based on interaction score: 0.97 (green line), 0.98 (purple line), 0.99 (blue light line), 1.0 (red line).

A total of 1,268 proteins identified in the core genome of *L. delbrueckii* probiotics strains were classified by SurfG+ software as cytoplasmic (CYT) (n = 918), membrane (ME) (n = 204), protein surfaces exposed (PSE) (n = 105), and secreted (SE) (n = 41) (Figure 4A). Of these proteins, 22 classified as secreted, 17 PSE, eight cytoplasmic, and two membranes were predicted by SPAAN with a high probability of being an adhesin (score >0.7) (Figure 4B), including LysM peptidoglycan-binding domain-containing protein (LysM), aggregation promoting protein (Apf), proteinase B (PrtB), penicillin-binding protein (Pbp1A), oligopeptide ABC transporter (OppA), lipoteichoic acid synthase (LtsA), phosphoglycerate mutase (Pgm1), peptide methionine sulfoxide reductase (MsrA), fluoride efflux transporter CrcB (CrcB), among others (Supplementary Table S6).

The functional characteristics of these 49 predicted adhesin-like proteins were determined using COG analysis. These proteins were spread over 13 COGs related to “cellular processes and signaling” (38.7%) (e.g*.,* cell wall/membrane/envelope biogenesis—28.6%; cell motility—2%; intracellular trafficking, secretion, and vesicular transport—2%; cell cycle control, cell division, chromosome partitioning—4.1%; and post-translational modification, protein turnover, and chaperones—2%), “metabolism” (20.4%) (*e.g.,* amino acid transport and metabolism-10.2%; nucleotide transport and metabolism—2%; carbohydrate transport and metabolism—6.2%; and inorganic ion transport and metabolism—2%), and “information, storage and processing” (6.1%) (*e.g.,* transcription—4.1%; and translation, ribosomal structure, and biogenesis—2%). Furthermore, 33.3% of adhesin-like proteins were assigned to the “poorly characterized” category (function unknown) (Figure 4C).

The core proteins with a high adhesion profile were also evaluated to interact with human immune proteins. InterSPPI software predicted 44 interactions (Supplementary Table S7). The proteinase PrtB was the most frequent interaction among the core proteins. Other immunomodulatory proteins were also predicted, such as LysM peptidoglycan-binding domain-containing protein (LysM), lipoteichoic acid synthase (LtsA), penicillin-binding protein (Pbp1b), N-acetylmuramidase (Acm), putative lipoprotein A-antigen (TcsA), among others (Figure 4D), demonstrating that these proteins can be involved with immunoregulatory ability of the *L. delbrueckii* probiotics strains. Regarding human proteins, the nuclear factor NF-κB p105 subunit (NFKB1), engaged in TLR/NF-κB signaling pathway, was the most frequent interaction. However, other human immune proteins involved in TLR2/4-MAPK, TLR2/4-NF-κB, and NOD-like receptor signaling pathways also interacted with immunoregulatory proteins of *L. delbrueckii* strains, such as TLR4, TRAF6, RELA, NFKBIA, NOD1, NOD2, FOS, JUN, and MAPK10, among others (Figure 4D; Supplementary Table S7).

## Discussion

Comparative genomics revealed a high variation level in the genome of nine *L. delbrueckii* probiotics strains, with the subspecies *lactis* presenting a larger genome size (Mb) than subspecies *bulgaricus*, corroborating the findings of El Kafsi et al. (2014). This genomic variation can be related to the differences in the number of unique genes observed across the strains, in which the subspecies *lactis* had the highest number. The pangenome analysis of *Lactobacillus delbrueckii* species has already been carried out by Inglin et al. (2018) and Kim et al. (2021). However, the above authors did not perform a functional analysis related to the probiosis of these strains. Thus, in our work, the performance of a combined analysis of pan-genome data of nine potential *L. delbrueckii* probiotics strains allowed us to obtain more robust data related to the most relevant characteristics of the probiose of these strains, mainly related to their ability to survive the TGI, adhesion, antibacterial activity, and immunomodulation.

Functional analysis of the core genome revealed that the proteins of nine *L. delbrueckii* probiotic strains are mainly involved in genetic and environmental information processing and metabolic activities, which suggests the importance of these genes in conserved cellular processes of these microorganisms to survive and adapt to specific environments or host.

One of the first adaptation steps of probiotics to the host involved molecular/cellular mechanisms related to their response to GIT stressors (stomach acidity and bile salt) (Papadimitriou et al., 2016). The core genome of *L. delbrueckii* probiotics strains harbors genes related to these stress response mechanisms, mainly including transcriptional regulators expression (e.g., two-component sensor histidine kinase), proton extrusions, and bile efflux (e.g., Na+/H+ antiporter, F0F1 ATPase genes, glycine/betaine ABC transporter permease), metabolic response (e.g., acetate kinase, pyruvate oxidase, ornithine decarboxylase), and heat shock/chaperones proteins production (e.g., GroEL, GroES, DnaK, DnaJ, ClpX). The expression of these genetic factors can be essential to the survival strategy of these bacteria on the GIT, allowing them to arrive in viable amounts sufficient to promote their interactions and beneficial effects with the specific-host sites of action. Genome and phenotype-scale studies demonstrated that these survival and adaptation mechanisms were observed in *L. delbrueckii* LJJ (Li et al., 2020) strain and are also shared with others, such as UFV H2b20 (Ferreira et al., 2013), 2038 (Hao et al., 2011), ATCC 11842, and CNRZ327 (El Kafsi et al., 2014), and CIDCA 133 (De Jesus LCL. et al., 2021).

This study´s probiotics *L. delbrueckii* strains were mainly isolated from dairy products, supporting the prediction of core genome enzymes related to a conserved proteolytic and metabolic sugar system. This high metabolic property enhances the fermentation ability of these strains with the production of essential metabolites (e.g., bioactive peptides, lactate, SCFA, and vitamins). These compound’s synthesis requires specific enzymes (e.g., proteinases and peptidases, glyceraldehyde-3-phosphate dehydrogenase, pyruvate kinase, phosphoketolase, acetate kinase, lactate dehydrogenase, riboflavin kinase, thiamine pyrophosphokinase, among others) of these strains, involved in proteolysis, and both phosphoketolase or Embden-Meyerhof (EMP) metabolic pathways (Kandler, 1983; Ye et al., 2021).

A vital feature derived from the fermentation process by probiotic strains is their antimicrobial activity due to organic acids, hydrogen peroxide, and bacteriocins production. The genome of *L. delbrueckii* probiotics strains has genes coding for these antimicrobial compounds (e.g., bacteriocin enterolysin A, D-lactate dehydrogenase, L-lactate dehydrogenase, acetate kinase, L-lactate oxidase, glycolate oxidase, and pyruvate oxidase), which makes them highly relevant in the food industry, since when used in the fermentation of food dairy products, it can control and preserve these products against the food spoilage of pathogens. The antibacterial effect associated with these *L. delbrueckii*-producing compounds against some pathogens, such as *Salmonella sp.*, *Enterococcus faecalis*, *Escherichia coli*, *Gardnerella vaginalis*, *Listeria monocytogens*, *Pseudomonas aeruginosa*, has been previously reported (Evivie et al., 2020; De Jesus LCL. et al., 2021; Qiu et al., 2021).

It is essential to identify genes/metabolic pathways and characterize bioproducts produced by probiotic bacteria with high fermentative capacity since studies have demonstrated the beneficial effects of fermented products derived from these microorganisms in GIT inflammatory diseases. For example, milk fermented by *L. delbrueckii* CNRZ327 (2 × 10^9^ CFU/mL) attenuated TNBS-induced colitis in a murine model, improving the epithelial architecture, and reducing inflammatory parameters (IL6, TNFα, MPO) and oxidative markers (COX2 and Hmox) (Plé et al., 2016). Similar effects were reported in *L. delbrueckii* CIDCA 133, whose milk fermented by the strain preserved the intestinal epithelium from the inflammatory damage caused by the chemotherapy drug 5-FU (300 mg/kg) (De Jesus et al., 2019). Another study demonstrated that intake of yogurt fermented with *L. delbrueckii* 2038 improves aging by metabolites production and microbiota and intestinal epithelial regulation (Usui et al., 2018). It is suggested that these effects can be associated with the production of organic acids (lactate, SCFA) and bioactive metabolites (vitamins) produced by these bacteria, although these studies did not assess their concentration. However, it is important to highlight that the ability of *L. delbrueckii* species to produce SCFA or vitamins with host health benefits has been previously reported (Laiño et al., 2012; Levit et al., 2018; Dan et al., 2019), makes them promise to be used as an adjuvant for the treatment of inflammatory GIT diseases and other pathological conditions due to their reported antioxidants, anti-inflammatory, and immunomodulatory properties.

Immunomodulatory and anti-inflammatory properties or probiotics bacterial can also be related to the surface layer proteins or extracellular proteins (Hidalgo-Cantabrana et al., 2020; Chandhni et al., 2021) due to the ability of these proteins to interact with the host cells via pattern recognition receptors (e.g., Toll-like receptors-TLR, NOD-like receptors-NLR) inducing specific signalization pathways responses, as nuclear factor kappa B (NF-κB) and mitogen-activated protein kinase (MAPK) (Delgado et al., 2020)*.* This hypothesis is corroborated by Rocha et al. (2012) when they showed that surface-exposed proteins of the *L. delbrueckii* CNRZ333 strain played a role in NF-κB immune modulation (Rocha et al., 2012).

*L. delbrueckii* strains characterized as probiotics shared genes with potential interaction with inflammatory pathways-related human immune proteins, including proteinase B (PrtB), penicillin-binding protein (Pbp1A), and lipoteichoic acid synthase (LtsA), among others, with PrtB being the most interacting protein. PrtB is a cell envelope-associated serine protease essential to milk casein degradation (Gilbert et al., 1996). The expression of this protein and its analogs producing bioactive health-beneficial peptides has been suggested to be crucial to the immunomodulatory properties of *L. delbrueckii* strains (De Jesus LCL. et al., 2021). For example, De Jesus LCL. et al. (2021) showed that predicted proteins of *L. delbrueckii* CIDCA 133 with a high adhesin profile, including PrtB protein, interacted with human immune proteins involved with NF-κB signaling pathway activation. These findings can be related to their *in vivo* results. It was demonstrated that consumption of this probiotic strain presented an anti-inflammatory profile by activating TLRs receptors (*Tlr2, Tlr4*), decreasing *Nfkb1* and enhancing immunoregulatory markers *Il10* and *Tgfb* gene expression (De Jesus LCL. et al., 2021). These results are also supported by Espeche Turbay et al. (2012). They demonstrated that milk β-casein degradation by *L. delbrueckii* CRL581 ameliorates TNBS-induced acute intestinal inflammation by increasing immunoregulatory IL10 and decreasing leukocytes infiltrate and the IFNγ pro-inflammatory marker (Espeche Turbay et al., 2012). It is believed that these effects can be attributed to its cell envelope-associated proteinase PrtL activity (Villegas et al., 2015).

Anti-inflammatory properties of other surface layer components of *L. delbrueckii* strains have also been reported as extracellular polysaccharides of *L. delbrueckii* TUA4408L, which presented antiviral activity against rotavirus infection in porcine cells by modulating TLR2/4, interferon regulatory factor (IRF)-3, and the antiviral factors IFN-β, MxA, and RNase L expression (Kanmani et al., 2018). Altogether, these findings reveal that these bacteria factors are essential to leading the biological process of the host, mainly immune regulation. Therefore, based on these findings, the knowledge at the genomic level of the individual characteristics of probiotics, as well as the genetic factors associated with their immunoregulatory capacity, can facilitate individualized or personalized use of them for clinical applications, thus being an alternative approach to the problems arising from the use of live beneficial microorganisms in clinical practice. Furthermore, the exploration of genetic factors can contribute to validating the role of these probiotics-derived bioactive molecules in different pathological conditions, including their beneficial effects on those that affect distant sites and organs (e.g., skin, respiratory and urogenital tracts, brain, bones, among others) (Reid et al., 2017; Bubnov et al., 2018). Thus, we reinforce that further studies, including knockout genes or heterologous production of these proteins, must be performed to validate these genotypic findings with the phenotypic reported results described for these strains and elucidate the underlying mechanisms involved in their immunomodulatory activities.

In summary, this first probiotic genomic characterization study for potential *L. delbrueckii* probiotics species shows that these bacteria share a broad gene repertoire that functionally may be responsible for phenotypic features attributed to these strains on the host. The data presented support other studies that aim to identify genetic factors and mechanisms related to the beneficial effects of new probiotic targets from the *Lactobacillus* species with high commercial and biotechnological relevance. Furthermore, these data open perspectives for new studies to be carried out to evaluate the predicted interacting bacteria proteins with human immune proteins as possible anti-inflammatory molecules to be tested in therapeutic approaches to different inflammatory conditions.
